# Lipids from the purple and white açaí (*Euterpe oleracea* Mart) varieties: nutritional, functional, and physicochemical properties

**DOI:** 10.3389/fnut.2024.1385877

**Published:** 2024-07-17

**Authors:** Orquídea Vasconcelos Santos, Yasmin Silva Lemos, Leyvison Rafael Viera da Conceição, Bárbara E. Teixeira-Costa

**Affiliations:** ^1^Programa de Pós-Graduação em Ciência e Tecnologia de Alimentos, Instituto de Tecnologia, Universidade Federal do Pará (UFPA), Belém, Pará, Brazil; ^2^Programa de Pós-Graduação em Engenharia Química, Instituto de Ciências Exatas, Universidade Federal do Pará (UFPA), Belém, Pará, Brazil; ^3^Programa de Pós-Graduação em Biotecnologia, Universidade Federal do Amazonas (UFAM), Manaus, Amazonas, Brazil; ^4^Departamento de Nutrição e Dietética, Faculdade de Nutrição Emília de Jesus Ferreiro, Universidade Federal Fluminense (UFF), Rio de Janeiro, Brazil

**Keywords:** vegetable oils, *Euterpe oleracea*, Amazonian fruit, tropical fruit, superfruit, fatty acids profile, açaí, assaí

## Abstract

The Brazilian superfruit called Açaí or Assaí has gained interested from researcher and consumers worldwide, due to its health-related properties. In this context, this pioneering study aimed to compare the physicochemical, nutritional, and thermal properties of vegetable oils obtained from two varieties of açaí (*Euterpe oleracea*), purple and white. Both açaí oils from white (WAO) and purple (PAO) varieties were obtained by using the conventional solid–liquid extraction, which resulted in oil yields ranging from 52 to 61%. WAO and PAO were analyzed by their edibility quality parameters given the recommendations from Codex Alimentarius; their nutritional functionality indices and their composition of fatty acids and triglycerides content were estimated. Both oils showed low levels of acidity and peroxides, <1.8 mg KOH g^−1^ and < 1.7 mEq kg^−1^, respectively, which are good indicators of their preservation status, agreeing with the food regulations. PAO and WAO showed differences among the composition of fatty acids, mainly related to the content of monounsaturated fatty acids (MUFAs), which were 62.5 and 39.5%, respectively, mainly oleic acid. Regarding the polyunsaturated fatty acids (PUFAs), the WAO showed up to 23% of linoleic acid, whereas the PAO exhibited up to 11% of it. These differences reflect on the values of the nutritional functionality indices, atherogenic (AI), thrombogenic (IT), and hypocholesterolemic/hypercholesterolemic ratio (H/H). Both PAO and WAO showed low levels of AI and TI and superior values of H/H than other oilseeds from the literature. These results indicate the nutritional properties of açaí oils regarding a potential cardioprotective effect when included in a regular dietary intake. The thermogravimetric behavior and the evaluation of oxidation status by infrared spectroscopy (FTIR) were also studied. Both açaí oils demonstrated higher thermal stability (with an onset temperature ranging from 344 to 350 °C) and low indications of oxidation status, as no chemical groups related to it were noted in the FTIR spectrum, which agrees with the determined acidity and peroxide content. Moreover, the FTIR analysis unveiled characteristic chemical groups related to fatty acids and triglycerides, agreeing with the literature reports. These findings collectively contribute to a deeper comprehension of the nutritional and functional properties between white and purple açaí oils, offering valuable insights into their potential health, food, and industrial applications.

## 1 Introduction

The so-called superfruits or superfoods have gained popularity among researchers and consumers. Among this, açaí berries stand out as an emerging Amazonian ‘superfruit’, mainly because of their nutritional and health-related properties (1). The higher concentration polyphenols, with prominent two groups of anthocyanins, cyanidin-3-glycoside and cyanidin-3-rutinoside, are linked to their antioxidant, anti-inflammatory, and other therapeutic properties (1–4). Diverse studies have demonstrated the health benefits of the intake of açaí (2, 4–6). These beneficial properties have led to a growing popularity of açai all over the world, especially in the USA, Europe, and Japan (7).

Açaí palm tree (*Euterpe* genus) belongs to the *Arecaceae* family, and its fruits exhibit a globular or ovoid drupaceous form that ranges from 1 to 2 cm in diameter and 0.8–2.3 g of weight (1). The fruit epicarp is firmly adhered to its mesocarp, which is the edible portion that represents a thin pulp layer approximately 1–2 mm (8). In Brazil, three species of açaí can be found, *Euterpe oleracea*, *Euterpe precatoria*, and *Euterpe edulis* (1). The most consumed and produced açaí fruit belongs to the *Euterpe oleracea* species and is linked to its dark purple color, which is mainly given by the presence of anthocyanins (1, 8). The white açaí variety is commonly found in street markets and food stores and consumed in the Northern region of Brazil, especially within the state of Pará (which is also the largest açaí producer) (8). Data provided by the Federation of Industries of the State of Pará (FIEPA) reveal that the state exported over 8.158 million tons of açaí in 2022, with a financial trade of over US$16.5 million (R$133.8 million) (9).

Many physicochemical and nutritional differences between the three species of Brazilian açaí have been reported (1, 10–12). Those differences are related not only to the characteristics of each specie but also to other environmental factors, such as climate, harvesting season, geographical location, and many others. Because of these, açaí fruits from the same species but harvested from different geographical locations can show diverse physicochemical and nutritional properties. Generally, açaí fruits from the *Euterpe oleracea* species possess a high content of lipids approximately 50%, fibers with up to 25%, and proteins with up to 10%, which makes it a highly caloric food, especially because the lipids content (1). This lipid content is composed of high levels of unsaturated fatty acids, especially oleic acid, making açaí oil considered nutritionally similar to olive oil (13). Despite the high content of monounsaturated fatty acids (MUFA) with up to 60.6%, açaí oil from *Euterpe oleracea* specie also shows up to 13.3% of polyunsaturated fatty acids (PUFA), contributed significantly by linoleic acid (12.5%), and up to 26.1% of saturated fatty acids, from which palmitic acid stands out (13). Regarding the content of bioactive substances, Matta et al. (8) have studied the polyphenols content in the white açaí and found that the pulp fruit displays total phenolics ranging up to 11.70 ± 0.24 mg gallic acid equivalent/g, while a total flavonoid was approximately 2.38 ± 0.35 mg quercetin equivalent/g, and less than <0.01 mg cyanidin-3-glucoside equivalent/g as total anthocyanins. These substances play an important role in the preservation of the pulp fruit as well as show relevant biologically health properties incorporated into the dietary intake (2, 14–16). As far as it is known, there is scarce information available regarding the physicochemical and nutritional properties of the white variety of açaí, compared to the common purple açaí fruit.

In this context, this study aimed to study the physicochemical, nutritional, and thermal properties of vegetable oils obtained from two varieties of açaí (*Euterpe oleracea*), purple and white. For this, both açaí oils were analyzed by their edibility quality parameters given the recommendations from Codex Alimentarius, their nutritional functionality indices and the composition of fatty acids and triglycerides content were estimated. The thermogravimetric property and the evaluation of oxidation status by infrared spectroscopy (FTIR) were also studied.

## 2 Methodology

### 2.1 Materials and methods

The pulp of açaí from the purple (PAO) and white (WAO) varieties were purchased at a commercial shop market located in the city of Belém, Pará, Brazil, duly certified by the national sanitary surveillance agency. This study was registered in the Brazilian National System for the Management of Genetic Heritage and Associated Traditional Knowledge (SisGen) for the research use of this vegetal product from the Brazilian Flora (registration number—AF69F86). The pulp samples were transported in polyethylene cartons and styrofoam boxes and kept under refrigeration conditions until the Food Sciences laboratory at the Faculty of Nutrition (FANUT), Federal University of Pará (UFPA), where it was immediately frozen at −18°C ± 2°C in conventional freezer equipment. After this, the açaí pulp samples were freeze-dried at −40°C for 48 h under vacuum using a Solab freeze-dryer (model SL-404, Solab Científica, Piracicaba—SP, Brazil). The freeze-dried samples were vacuum-packed and stored under light protection at room temperature until further analysis. All the chemicals used in this study were of analytical grade purchased from Sigma-Aldrich Brazil Ltd. (São Paulo, SP, Brazil).

#### 2.1.1 Oil extraction and yield

The açaí oil extraction was performed using the solid–liquid method carried out on a Soxhlet apparatus, according to the method n° 948.22 of the Association of Official Agricultural Chemists—AOAC International (17), using *n*-hexane as a solvent extractor. The extraction procedure was performed in triplicate for each açaí variety, purple and white. The extracted oils were named WAO and PAO, regarding the white and purple açaí samples, respectively. The oil extraction yield (OY%) was calculated according to the Eq. 1.


(1) $$OY\%=\frac{W_{\text{oil}}}{W_{\text{sample}}}\times 100$$

Where W_oil_ is the weight of extracted oil (in grams), and W_sample_ is the weight of freeze-dried açaí pulp (in grams).

### 2.2 Physicochemical parameters of açaí oils

To evaluate the physicochemical quality of the açaí oils, WAO and PAO, as a source of edible lipids, their density, refractive index, and acidity and peroxide values were performed according to American Oil Chemists’ Society (AOCS) official methods. Density was measured using a digital density meter (DA-130; Kyoto Electronics Manufacturing Co., Ltd., Kyoto, Japan) at room temperature (25°C), and the refractive index was measured at 20°C using an Abbe refractometer (model AR/200, Tecnal, Piracicaba—SP, Brazil) according to the official method of AOCS (18). Acidity and peroxide values of the açaí oils were determined according to official methods Cd 3d-63 and Cd 8–53 from AOCS, respectively (19).

### 2.3 Determination of fatty acids profiles of açaí oils

The fatty acids (FAs) profile of WAO and PAO were determined by gas chromatography (GC) using the following methodologies. First, the FAs were methyl esterification according to the boron trifluoride (BF_3_) method ISO 5509:2000 reported by the International Standardization Organization (ISO) (20). After phase separation, the supernatant was collected and used in gas chromatography (GC Varian 430) analysis to determine the fatty acid profile according to ISO 5509 (20). After phase separation, the supernatant was collected and submitted to GC analysis. The GC was performed using a gas chromatography equipped with a microcomputer using the software Galaxie Chromatography based on the following chromatographic conditions: fused silica SP®-2560 capillary column (Supelco, United States) (100 m in length x 0.25 mm of internal diameter) containing 0.2 μm of polyethylene glycol. The operating conditions were as follows: split injection, ratio of 50:1; column temperature at 140°C for 5 min, programmed with an increasing rate of 4°C per min up to 240°C, carrier gas: helium, isobaric pressure of 37 psi, the linear velocity of 20 cm/s; makeup gas: helium at 29 mL/min; injector temperature of 250°C, model Varian CP-8410 (Autosampler); detector temperature of 250°C. The qualitative composition was determined by comparing the time of peak retention with the respective profiles of fatty acids. Internal standards, C_15:0_—methyl pentadecanoate and 37-Component FAME Mix (methyl esters of fatty acids ranging from C_4_ to C_24_ CRM47885 from Sigma–Aldrich, Milan, Italy) were used. The quantitative composition was carried out by area normalization, being expressed in mass percentage as established by the official method Ce 1–62 (21).

### 2.4 Nutritional functionality of lipid fractions

The composition of FAs of WAO and PAO was classified into fractions, as saturated fatty acids (SFA), unsaturated fatty acids (UFA), monounsaturated fatty acids (MUFA), and polyunsaturated fatty acids (PUFA), according to the presence and number of double or triple bonds. These fractions were used to determine the nutritional indices as follows: atherogenicity index (AI), thrombogenicity index (TI) according to Ulbricht and Southgate (22), and the hypocholesterolemic/hypercholesterolemic ratio (HH) as proposed by Chen et al. (23). The Eqs 2–4 were used to calculate the AI, TI, and HH indices, respectively.


(2) $$\text{AI}=\frac{\left[\left(C_{12:0}+\left(4\times C_{14:0}\right)+C_{16:0}\right)\right]}{\left(\text{PUFA}+\text{MUFA}\right)}$$

(3) $$TI=\frac{\left(C_{12:0}+C_{16:0}+C_{18:0}\right)}{\left[\begin{array}{l} \;\left(0.5\times \text{MUFA}\right)+\left(0.5\times n6\text{PUFA}\right)+\left(3\times n3\text{PUFA}\right)\\ +\left(\frac{n3\text{PUFA}}{n6\text{PUFA}}\right) \end{array}\right]}$$

(4) $$H/H=\frac{C_{18:1}+\text{PUFA}}{\left(C_{12:0}+C_{14:0}+C_{16:0}\right)}$$

### 2.5 Estimation of triacylglycerol composition of açaí oils

The triacylglycerol composition of açaí oils, WAO and PAO, were estimated using the open-access software PrOleos® (available online at https://lames.quimica.ufg.br/p/4035-courseware). This platform uses the hypothesis of 1,3-random-2-random distribution, thereby predicting the molar percentage of triacylglycerols (TAGs) present in the oil according to its fatty acid composition (24). Groups of TAGs with the same equivalent carbon number (ECN) and groups with less than 0.5% (w/w) of the total concentration were disregarded.

### 2.6 Fourier transform infrared spectroscopy (FTIR)

Fourier transform infrared spectroscopy (FTIR) analyses were carried out using a Perkin Elmer spectrometer, Frontier 98,737 model (Waltham, MA, United States) at ambient temperature in the 4,000–400 cm^−1^ range. The spectra were registered by averaging 20 scans with a resolution of 4 cm^−1^ in transmission mode. The sample WAO and PAO were analyzed as potassium bromide (KBr) disks.

### 2.7 Thermogravimetric analysis and differential calorimetric analysis (DSC)

The thermogravimetric analysis (TG) was used to investigate the thermal stability of WAO and PAO and was carried out for the samples under a nitrogen atmosphere on a TA Instrument, model Q-500 (New Castle, DE, United States). Approximately 10 mg of sample was heated from 25°C to 700°C at a 10°C/min rate. The derivative (DTG) curves were used to measure and compare the peak temperatures. Experimental data were analyzed with Origin 8.0 (OriginLab Corp., Northampton, MA).

The thermal properties of SRO were investigated using a DSC Q-1000 Differential Scanning Calorimetry (DSC) equipment from TA Instruments (New Castle, DE, United States). Samples of 5.0 ± 0.5 mg were sealed in an aluminum pan with a pinhole and subjected to a nitrogen atmosphere at a 50 mL min^−1^ flow rate. The first ramp was equilibrated at −50°C and heated until 250°C at a 10°C min^−1^ rate. An isothermal ramp kept the temperature at 250°C for 1 min, and then, a cooling ramp (quenching) at a fast cooling rate used to equilibrate the temperature at −50°C. All consecutive measurements were heated, cooled, and reheated from −50 to 250°C at a 10°C min^−1^ rate. The DSC profile was analyzed with the Universal Analysis software version 4.2 (TA instruments, New Castle, DE, United States).

### 2.8 Statistical analysis

The analysis of the oil yield, quality, and fatty acid profile was performed in triplicate (mean ± standard deviation), and the results were subjected to analysis of variance (ANOVA) at the significance level of 5% and Tukey’s test (*p* ≤ 0.05), using the software Statistica version 7.0.

## 3 Results and discussion

The results from the extraction and physicochemical characterization of the açaí oils, WAO and PAO, are presented in the following sections.

### 3.1 Extraction yield and physicochemical parameters of açaí oils

The extractions resulted in oil yields of 52.2% from the white açaí variety and 60.7% from the purple açaí variety. These results are higher than the findings of Buratto et al. (25) and are quite similar to the findings of Lucas et al. (26). Oliveira and Schwartz (10) cited that açaí fruits can possess from 21 to 53% of lipids. It is well known that conventional solid–liquid extractions, such as the Soxhlet-based ones, are frequently used to extract lipids from diverse food matrixes. However, other unconventional methods have been used for the same end. In the study by Silva et al. (13), the oil from freeze-dried açaí was extracted by using CO_2_ supercritical fluid extractions, and their oil yield ranged from 49.28 to 57.06%, with the following condition procedures: 60°C of temperature and 420 bar of pressure, and 70°C and 490 bar of pressure. These results show that açaí oil has a high extraction yield under diverse methods. The physicochemical quality parameters in the oils, WAO and PAO, are shown in Table 1.

**Table 1:** Physicochemical quality parameters in WAO and PAO.

Analyses	WAO	PAO	Recommended levels*
Acidity index (mg KOH/ g)	1.53 ± 0.15	1.75 ± 0.03	4.0 mg
Peroxide index (mEq/ kg)	1.43 ± 0.75	1.72 ± 0.75	15
Density (25°C g/ mL)	0.95 ± 0.07	0.94 ± 0.03	ND
Refractive Index (at 25°C)	1.457 ± 0.00	1.477 ± 0.00	ND

WAO, white açaí oil; PAO, purple açaí oil. Data are presented as mean ± standard deviation (*n* = 3). ^*^Recommended levels by Codex Alimentarius (27). ND, Not defined.

Table 1 presents the results from the physicochemical quality indices for both white and purple açaí oils. The acidity and peroxide content showed lower levels than the Codex Alimentarius (27) recommendation for edible vegetable oils. Acidity and peroxides can be used as indicators for lipid oxidation and hydrolysis, which should be as low as possible in vegetable oils for human consumption (28, 29). Higher values of acidity can be correlated with a higher content of free fatty acids from hydrolytic degradation, while higher peroxide values indicate the presence of primary oxidation products and hydroperoxides, which can be decomposed into other secondary oxidation products (17, 28). It is well known that several environmental and processing factors can influence the quality of vegetable oils, including ripening state, harvesting season, post-harvesting conditions, and processing steps, such as extraction methods, as well as inner factors related to the fruit species.

As both, WAO and PAO showed low levels of acidity and peroxides, it is possible to suggest that both oils can be edible and that the extraction method and other inner factors related to açaí pulp compounds, such as polyphenols, could contribute to preserving their quality. In the study by Pacheco-Palencia et al. (18), açaí oil displayed low free fatty acids (<0.1%) and low peroxide values (<10 mEq/kg) prior to and after 10 weeks of storage at 20, 30, or 40°C. These authors suggest that the content of phenolic substances in açaí oil was able to protect it from oxidative degradation under storage conditions (18). In another work, açaí oil displayed low peroxide values (<10 mEq/kg) up to 3 days of oxidative accelerated storage conditions, 60°C for 7 days (19). When the açaí oil was incorporated with 400 ppm of myricetin, the low levels of peroxides increased to 5 days under 60°C of storage (19).

The density and refractive index of both açaí oils, PAO and WAO, were quite similar, ranging from 0.94 to 0.95 g/mL and approximately 1.48 and 1.46, respectively. Castro et al. (20) found that açaí (*Euterpe oleracea* Mart.) oil extracted by using a solid–liquid extraction with a Soxhlet apparatus showed a density value approximately 0.76 g/mL, a lesser value than this study. These differences can be related to the inner factors of açaí samples as well as to the chemical composition of the extracted oils. The apparent density of vegetable oils is relevant information regarding their flow rate as well as a mass transfer during processing, especially during frying and cooling steps when cooking (21). Despite this, both parameters are important physical characteristics used in the global vegetable oil trade. Apparent density is relevant information for the exportation of vegetable oils because the volume must be converted into mass while loading and discharging of a ship (22). The Codex Alimentarius (27) presents the apparent density and refractive index for some vegetable oils, such as palm oil and palm kernel olein, which range from 0.889 to 0.895 g /mL (at 50°C) and 0.904 to 0.907 g /mL, and 1.454 to 1.456 (at 50°C) and 1.451 to 1.453, respectively.

### 3.2 Fatty acids (FAs) profiles of açaí oils

The FAs profile of both açaí oils, WAO and PAO, are presented in Table 2. The monounsaturated fatty acids (MUFAs) were dominant in PAO and WAO, especially due to the major content of oleic acid, ~62.5% and ~ 39.1%, respectively. It was noted that the WAO showed higher levels of saturated fatty acids (SFAs), ~37.1%, than the PAO, ~23.8%. The main predominant SFAs in both oils were palmitic acid followed by stearic acid. Regarding the content of polyunsaturated fatty acids (PUFAs), the WAO showed higher levels than PAO, especially regarding linoleic acid (C18:2 ω-6). The higher content of oleic acid in PAO could be a suggestion that it could have a greater nutritional property than WAO, as diets with a higher proportion of UFAs than SFAs have been linked with a reduction of total cholesterol and the prevention of cardiovascular diseases (23). The açaí oils studied by Silva et al. (13) displayed a similar content of MUFAs, which ranged from 65.43 to 67.72%, also related to the major proportion of oleic acid. These authors also found that palmitic acids were the main SFAs, ranging from 21.15 to 21.79% (13).

**Table 2:** Fatty acid profile in PAO and WAO.

Fatty acids	PAO (%)	WAO (%)
Saturated fatty acids (SFAs)		
Myristic acid (C:14)	N.d.	0.11 ± 0.03
Palmitic acid (C:16)	21.89 ± 1.33^a^	31.08 ± 0.33^b^
Stearic acid (C18:0)	1.86 ± 0.57^a^	5.73 ± 0.59^b^
Arachidic acid (C20:4)	N.d.	0.62 ± 0.07
Behenic acid (C20:0)	N.d.	0.19 ± 0.05
Monounsaturated fatty acids (MUFAs)		
Palmitoleic acid (C16:1)	3.06 ± 0.7^a^	0.39 ± 0.08^b^
Oleic acid (C18:1 ω-9)	62.45 ± 3.07^a^	39.08 ± 3.23^b^
Polyunsaturated fatty acids (PUFAs)		
Linoleic acid (C18:2 ω-6)	10.26 ± 1.13^a^	22.80 ± 2.03^b^
Linolenic acid (C18:3 ω-3)	0.49 ± 0.13	N.d.
Σ Saturated fatty acids (%)	23.75	37.11
Σ Unsaturated fatty acids (%)	76.256	62.890
Σ Monounsaturated (%)	62.453	39.465
Σ Polyunsaturated (%)	10.746	22.803
Σ ω-6 (%)	10.258	22.803
Total (%)	99.998	99.996

WAO, white açaí oil; PAO, purple açaí oil; Data represent mean ± standard deviation (*n* = 3). The same letters on the same line indicate that there are no significant differences.

Comparatively, the PAO showed higher levels of oleic acid than the soya bean oil (17–30%), sesame seed oil (34.4–45.5%), palm oil (36.0–44.0%), sunflower seed oil, and rice bran oil (38–48%), and content of linoleic oil in the range of the palm olein (10.0–13.5%), and hazelnut oil (5.2–18.7%), and higher than coconut oil (1.4–6.6%) as presented in the Codex Alimentarius (27). The PAO showed greater fatty acid composition than palm oil (*Elaeis guineensis*), especially regarding the content of oleic acid. This information is relevant as the use of palm oil in diverse food products has grown exponentially, due to the improvement of sensorial properties in products incorporated with palm oil (24, 29). The dietary intake of UFAs, especially PUFAs from the omega-6 and omega-3 series, plays a significant role in the synthesis of key molecules to the immune system, such as eicosanoids and docosanoids (16, 30). These eicosanoids are transformed into prostanoids by cyclooxygenases or form leukotrienes by lipooxygenases, which will influence different cellular functions and influencing metabolic, physiological, pathological, and inflammatory processes in the body (16, 31). In this context, the consumption and use of vegetable oils with a greater content of unsaturated fatty acids should be pursued by the food industries and consumers, which could be an opportunity for the use of açai oils as ingredients in diverse food applications.

### 3.3 Nutritional functionality of lipid fractions

The nutritional functionality of lipid fractions in both açaí oils, PAO and WAO, is shown in Table 3. The P/S ratio can be related to a greater proportion of PUFAs in the oils, which are linked to the prevention of an increase of body weight in diets with high-fat intake (32). PAO displayed a higher P/S ratio than WAO, which is related to the higher amount of PUFAs in the first oil. PAO showed a similar P/S ratio to extra-virgin olive oil (0.6) and was higher than palm oil (0.02) (29). Açaí oils in the study by Silva et al. (13) displayed a P/S ratio ranging from 0.41 to 0.56. Beyond the P/S ratio, the AI, TI, and HH ratios are also relevant indices related to the nutritional function of FAs on human dietary intake. The first two indices, AI and TI, should be as low as possible, while the HH ratio should be higher (29, 33). WAO presented a minor value for the AI and TI indices, ~0.3 and ~ 0.6, respectively, and a higher proportion for the HH ratio, ~3.3. Compared to the palm oil (AI = 2.7 and TI = 3.5), both PAO and WAO, presented much lower values for AI and TI. The AI and TI values for the açaí oils studied in the study by Silva et al. (13) ranged from 0.28 to 0.29 and 0.52 to 0.54, respectively, quite similar values to those found in this study. Regarding the HH ratio, Silva et al. (13) presented values that ranged from 3.37 to 3.53.

**Table 3:** Nutritional functionality of lipid fractions in PAO and WAO.

Indices	PAO	WAO
P/S	0.614	0.452
AI	0.505	0.298
TI	1.183	0.627
HH	2.004	3.344

WAO, white açaí oil; PAO, purple açaí oil; P/S, polyunsaturated/saturated fatty acid ratio. AI, Atherogenicity Index; TI, Thrombogenicity Index; HH, Σ Hypocholesterolemic/Σ hypercholesterolemic.

In particular, the values of the HH ratio should be inversely proportional to the AI and TI indices because lower values of the last ones are an indication of their potential influence on cholesterol and low-density lipoprotein (LDL) in blood, which are correlated to atherosclerosis and coronary thrombosis (13). Moreover, the use of these parameters in the screening of dietary lipids with higher nutritional quality can be beneficial in the reduction of cardiovascular and other non-transmissible chronic diseases (34). Thus, it is possible to suggest that açaí oils could have cardioprotective effects when added to a regular dietary intake.

### 3.4 Estimation of composition of TGAs of açaí oils

Table 4 shows the estimated triacylglycerols composition of both açaí oils, PAO and WAO. Compounds that represented less than 0.5% of the total molecule content were not shown. Both oils showed different estimated content of TAGs. The predominant TAGs in the purple açaí oil were POO (C52:2) with 26.1% followed by OOO (C54:3) with 24.8% and OLO (C54:4) with 12.3%, while in white açaí oil, the main TAGs were PLO (C52:3) with 17.4%, POO (C52:2) with 14.9%, POP (C50:1) with 11.9% and OLO (C54:4) with 10.9%. In particular, these TAGs are composed of SU2 triacylglycerols. Silva et al. (13) found that the main TAGs in açaí oils were OOO, POO, OLiO, PLiO, and POP, with values ranging up to 27.97, 28.44, 15.15, 10.45, and 9.68%, respectively. These results show that the açaí oils studied display a quite similar composition of TAGs to the findings by Silva et al. (13), but eventual quantitative disparities can be related to the açaí origin, harvesting season, method of extraction, and other aspects. In this study, the TAGs with an equivalent carbon number of 54 (42.1%) predominated followed by the TAGs with an equivalent carbon number of 52 (41.8%) in PAO, while the TAGs with an equivalent carbon number of 52 (67.7%) were the main compounds, followed by the TAGS with 54 carbon number (30.7%). This shows that the PAO is predominantly composed of long-chain TAGs, which can contribute to reducing the risk of the occurrence of cardiovascular diseases linked to higher proportions of plasma cholesterol (35).

**Table 4:** Estimated triacylglycerols in PAO and WAO.

Triacylglycerol ECN	PAO (%Normalized)	Triacylglycerol ECN	WAO (%Normalized)
PPP (C40:0)	1.072	PPP (C40:0)	3.144
SPP (C50:O)	_	SPP (C50:0)	1.729
POP (C50:1)	9.160	POP (C50:1)	11.860
PLP (C50:2)	1.512	PLP (C50:2)	6.916
POPo (C50:2)	2.510	POPo (C50:2)	_
POS (C52:1)	1.589	POS (C52:1)	_
SOP (C52:1)	_	SOP (C52:1)	4.347
SLP (C52:2)	_	SLP (C52:2)	2.335
POO (C52:2)	26.101	POO (C52:2)	14.910
PLO (C52:3)	8.617	PLO (C52:3)	17.389
PoOO (C52:3)	3.575	PoOO (C52:3)	_
PLL (C52:4)	0.711	PLL (C52:4)	5.070
PoLO (C52:4)	1.180	PoLO (C52:4)	_
SOO (C54:2)	2.264	SOO (C54:2)	2.733
SLO (C54:3)	0.748	SLO (C54:3)	3.187
OOO (C54:3)	24.790	OOO (C54:3)	6.249
OLO (C54:4)	12.276	OLO (C54:4)	10.931
OLL (C54:5)	2.026	OLL (C54:5)	6.374
LLL (C54:6)	_	LLL (C54:6)	1.239

WAO, white açaí oil; PAO, purple açaí oil; ECN, equivalent carbon number; La, lauric acid; M, myristic acid; P, palmitic acid; Pa, palmitoleic acid; S, stearic acid; O, oleic acid; L, linoleic acid.

In the study by Almoselhy et al. (36), the TAGs from different olive oils were studied and they found that the OOO was the predominant TAG (30.32 to 32.90%), followed by POO with a content ranging from 26.45 to 28.36% and OOL ranging from 12.00 to 13.91%. Similarly, purple açaí oil displayed OOO and POO as the major TAGs. This is a good indication of the nutritional quality of the PAO compared to the olive oil.

### 3.5 Fourier transform infrared spectroscopy (FTIR)

**Figure 1:**
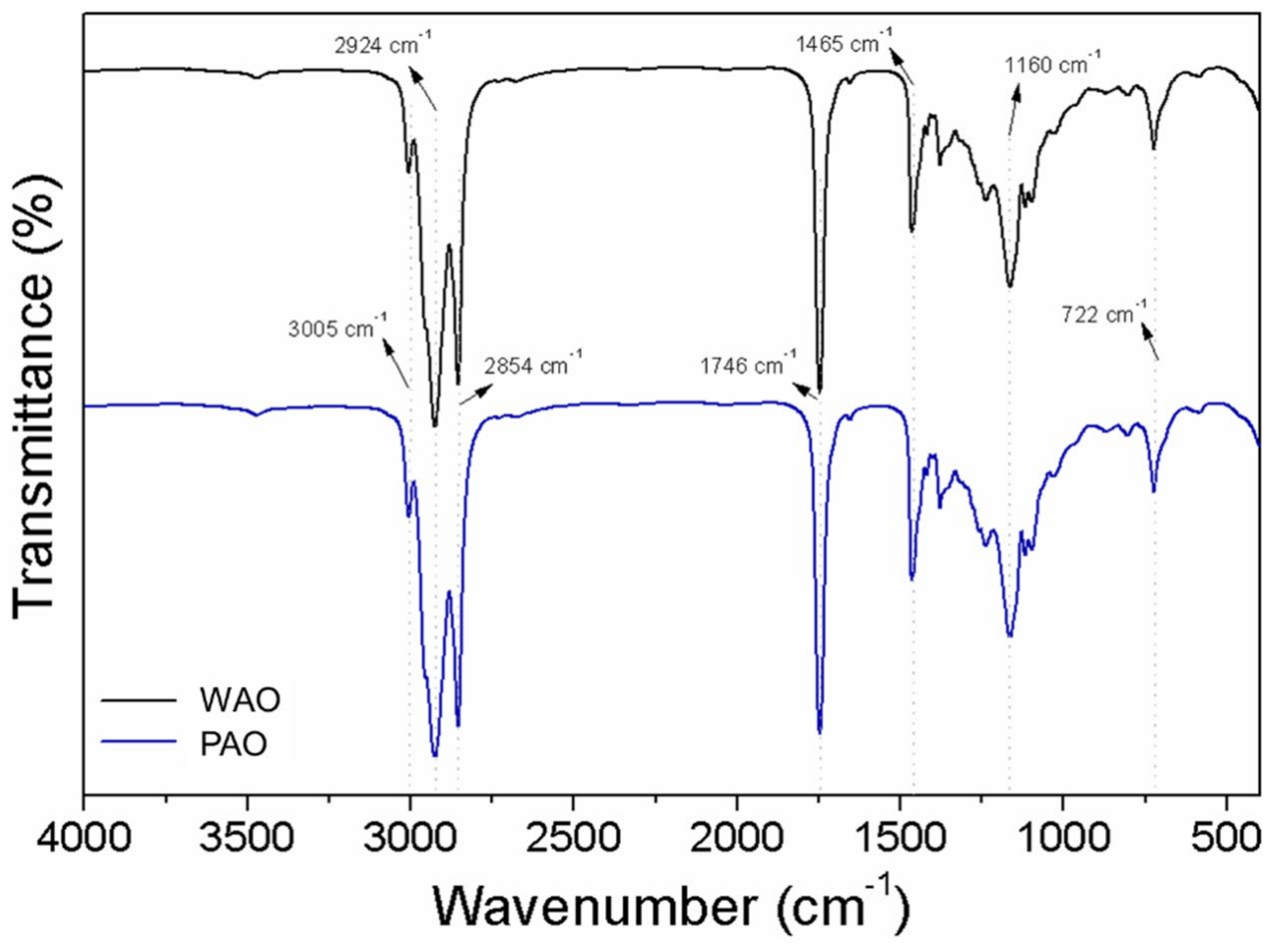
FTIR spectra of WAO (black line) and PAO (blue line).

The FTIR is an analytical technique used for the identification of some functional chemical groups of substances by their spectral bands and helps the evaluation of oxidation conditions, which can detect degradation and adulteration in vegetable oils (35, 37). The FTIR spectra of PAO and WAO are shown in Figure 1. The designation of bands was registered according to the literature. Both PAO and WAO displayed similar FTIR spectra with slight variations in the band frequencies and intensities, which could be due to their different composition and nature, especially FAs and other chemical substances.

The spectral patterns showed the presence of high-intensity bands related to triglyceride functional groups, approximately 3,005 cm^−1^ related to stretching vibration of (=C–H (*cis*)), at 2924 cm^−1^ to asymmetric stretching of (C–H) and 2,854 cm^−1^ linked to symmetric stretching vibrations of (–C–H (CH_2_)) (25, 35, 37). C18:2 fatty acids exhibit high frequency in these last band regions, which could be due to the presence of linolenic acyl and oleic acyl groups in both oils (38). The band approximately 1746 cm^−1^ can be related to C=O stretching vibrations of ester carbonyl functional groups (37, 39), while the bands approximately 1,465 cm^−1^ are associated with the bending vibrations (scissoring) of aliphatic CH_2_ and CH_3_ groups (–C–H) or stretching vibration of amino groups (N––C) (39–41). Both PAO and WAO presented significant amounts of oleic and linoleic acids, 40–60% and 10–23%, respectively; thus, it can be inferred that the high intensity of these bands approximately 1750 cm^−1^ may be linked to this lipid profile. Another prominent band ranging approximately 1,162 cm^−1^ can be linked to C–O stretching and C–H bending vibrations (29, 39). Fragoso et al. (42) found in their studies of grape phenolic compounds that the spectral regions from 1,133–1,457 cm^−1^ (1320–1,420 cm^−1^ due to the O-H bend) are related to signals of gallic acid, tannic acid, and (+)-catechin. Vanillic acid, syringic acid, protocatechuic acid, and other phenolic substances were quantified in açaí oil (*E. oleracea*) by Pacheco-Palencia et al. (18). The bands approximately 722 cm^−1^ are related to the bending vibrations of C–H (out of plane vibration of cis-disubstituted olefin) and saturated carbon–carbon bonds (29, 37, 39). Similar to this study, Teixeira-Costa et al. (41) found that açaí oil (*Euterpe oleracea*) also showed high-intensity bands in the regions 2,923 cm^−1^, 2,853 cm^−1^, 1744 cm^−1^, 1,160 cm^−1^, and 722 cm^−1^.

### 3.6 Thermogravimetric analysis

**Figure 2:**
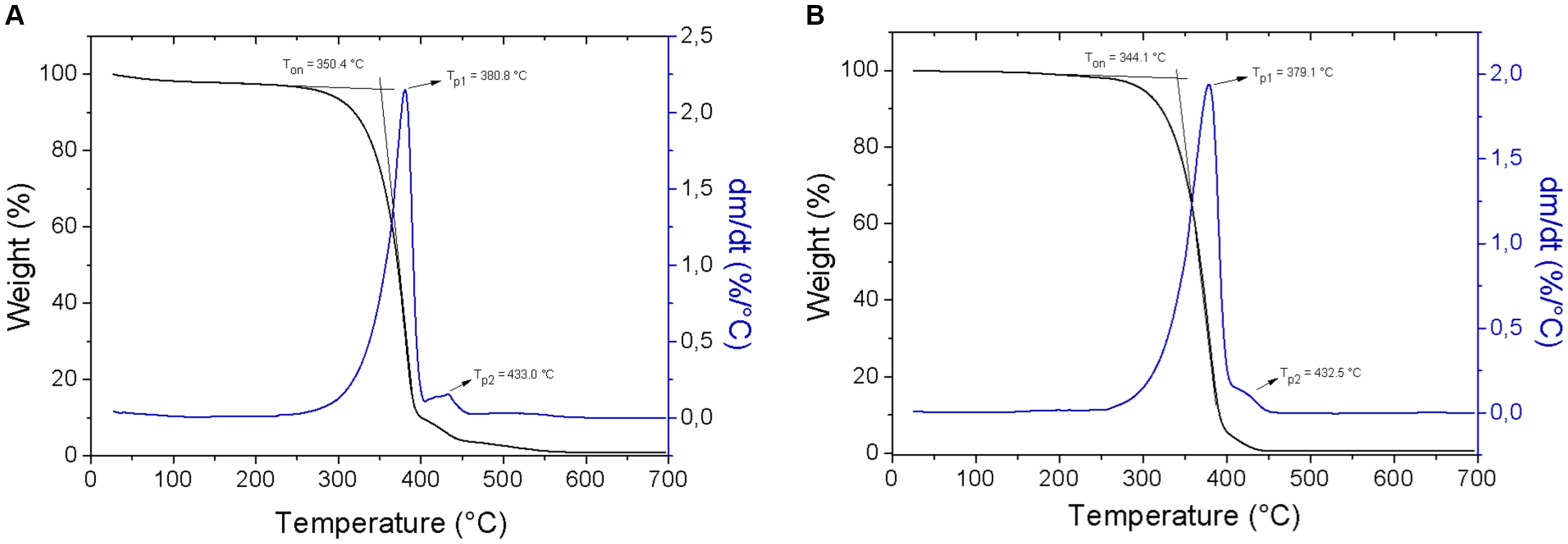
Thermograms (black line) and derivative curves (blue line) of **(A)** WAO and **(B)** PAO.

The thermogravimetric (TG) and differential thermogravimetric (DTG) curves of WAO and PAO are presented in Figure 2. The thermal degradation behavior of WAO and PAO occurred in one stage. For both samples, the initial decomposition temperature (T*_on_*) ranged approximately 344–350°C and the peak temperature (T*_peak_*) approximately 380°C. This result is similar to the T*_on_* values, 345°C, 342°C, and 340°C, found by Garcia et al. (43) on the studies with vegetable oils from Pequi pulp (*Caryocar brasiliense* Camb.), Baru (*Dypterix alata* Vog.), and Amburana (*Amburana cearensis* (Fr. Allem) A. C. Smith), respectively. Açaí oils showed slight differences in their thermal degradation study. The PAO presented the lowest thermal stability when compared to the WAO, probably due to the major amount of short-chain fatty acids, myristic (C:14) and palmitic acids (C:16).

The study of the thermal stability from the açaí oils, WAO and PAO, is listed in Table 5.

**Table 5:** Thermal stability of WAO and PAO.

Sample	T*_on_* (°C)	T*_peak_* (°C)	% W_Loss_	% Res_700°C_
WAO	350.4	380.8	99.1	0.9
PAO	344.1	379.1	99.4	0.6

WAO, white açaí oil; PAO, purple açaí oil.

Teixeira-Costa et al. (41) found that açaí pulp oil had a T*_on_* approximately 402°C and a T*_peak_* close to 440°C, which are superior temperatures from those found in this study. These differences can be related to the composition of fatty acids and oxidation status of açaí oil samples. Comparing the results of T*_on_* of palm oil, it is possible to note that both PAO and WAO seem more stable to thermal degradation, as the first showed temperatures of initial degradation ranging approximately 219–268°C (44). These results highlight the potential of incorporating açaí oils into diverse food applications, including those where higher temperatures are used, such as cooking or frying.

### 3.7 Differential scanning calorimetric (DSC) analysis

**Figure 3:**
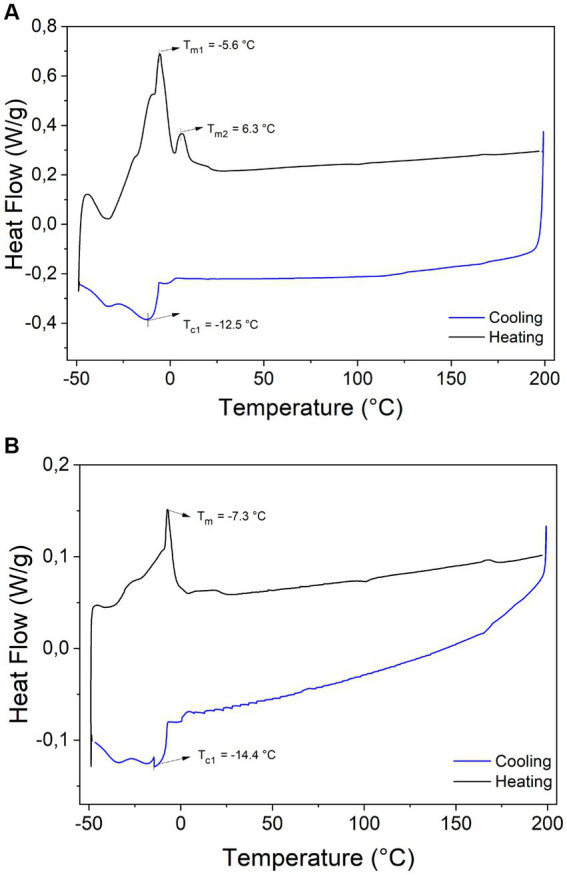
Heating (black line) and cooling (blue line) DSC curves of **(A)** WAO and **(B)** PAO.

The DSC thermograms after cooling and heating from −50°C to 200°C of WAO and PAO are shown in Figure 3. The crystallization studies are used to characterize the thermal behavior of fats and oils, which are highly related to their composition on FAs and TAGs and organization into polymorphic forms (45, 46). The DSC cooling thermograms of both açaí oils, WAO and PAO, showed only one exothermic peak at −12.5°C and − 14.4°C, respectively, related to the co-crystallization of the TAGs, which are mostly composed of unsaturated fatty acids and cooling in their uniquely exothermic region (46, 47). Lower peak temperatures can be linked to the properties of unsaturated fatty acids.

The thermogram of WAO (Figure 3A) shows a series of peaks named T_m1_ (−5.6°C) and T_m2_ (6.3°C) related to the most of unsaturated fraction to less unsaturated TAGs melting of the WAO (47), while the PAO showed only one peak at −7.3°C. The ∆*H* considered the enthalpy taken from the measurement of the area under the peaks, was 59.84 J g^−1^ and 6.65 J g^−1^ for WAO and PAO, respectively. The observed differences among PAO and WAO are related to their different content of FAs and TAGs. The ∆*H* of flaxseed oil during heating at 75°C ranged from 53.4 to 55.18 J g^−1^ (48). Other oilseeds, such as soybean and sunflower, show peak temperatures ranging from −15 to −25°C, due to their higher proportions of PUFAs (45).

Moreover, further investigations using X-ray diffraction or other methodologies that improve the discussion regarding the structural information agreeing with the DSC and TGA results, as well as the FTIR spectrum should be pursued, thus gaining more accurate information about açaí oils.

## 4 Conclusion

The present study showed that the white and purple açaí oils differ in many physicochemical parameters, as well as in their composition of FAs and TAGs, which influences their thermal stability and infrared spectra. The white açaí oil showed lower levels of acidity and peroxides than the purple açaí oil, which can be related to the greater susceptibility of the last one to oxidation status due to a higher proportion of unsaturated fatty acids. PAO showed greater contents of MUFAs (oleic acid), while the WAO displayed higher levels of PUFAs, especially linoleic acid. These differences in the content of FAs among both açaí oil samples influenced its estimated nutritional functionality indices and the composition of TGAs. When compared to palm oil, an important vegetable oil used in food products, both PAO and WAO showed better results in all nutritional functionality indices, which can indicate that the addition of açaí oil can contribute to the development of novel and healthier food products, due to its potential cardioprotective effect. The FTIR analysis indicated low levels of oxidation in both açaí oils, while the TGA and DSC analysis provided good data on thermal stability. Thus, this work showed that açaí oils have good physicochemical and nutritional qualities to be used as a functional ingredient in the development of novel food products.

## Data availability statement

The original contributions presented in the study are included in the article/supplementary material, further inquiries can be directed to the corresponding author.

## Author contributions

OS: Conceptualization, Data curation, Formal analysis, Funding acquisition, Investigation, Methodology, Project administration, Resources, Software, Supervision, Validation, Visualization, Writing – original draft, Writing – review & editing. YL: Investigation, Methodology, Writing – original draft. LC: Conceptualization, Investigation, Methodology, Writing – review & editing. BT-C: Conceptualization, Formal analysis, Investigation, Methodology, Writing – original draft, Writing – review & editing.
